# Drug-induced acute kidney injury: a cohort study on incidence, identification of pathophysiological mechanisms, and prognostic factors

**DOI:** 10.3389/fmed.2024.1459170

**Published:** 2024-10-29

**Authors:** Georgia Garcia, Vinicius Repetti Pacchini, Welder Zamoner, Andre Luis Balbi, Daniela Ponce

**Affiliations:** Hospital das Clínicas, Faculdade de Medicina de Botucatu, Botucatu, Brazil

**Keywords:** DI-AKI (drug-induced acute kidney injury), nephrotoxicity, prognosis, incidence, AKI (acute kidney injury)

## Abstract

**Introduction:**

Acute Kidney Injury (AKI) is a common clinical syndrome characterized by an abrupt decline in the glomerular filtration rate (GFR), which can cause severe alterations in blood volume and acid-base balance. Drug-Induced Acute Kidney Injury (DI-AKI) is associated with exposure to nephrotoxic medications, particularly among hospitalized patients. Adverse drug reactions comprises type A and type B reactions. Type A reactions are predictable based on the pharmacology of the substance, dose-dependent, and manifest as Acute Tubular Necrosis (ATN). Type B reactions are unpredictable, idiosyncratic, not dose-dependent, and manifest as Acute Interstitial Nephritis (AIN), Crystal-Induced Nephropathy, among others.

**Objective:**

To evaluate DI-AKI incidence, identify the main associated drugs and the pathophysiological mechanism of the observed injury, analyze prognostic factors associated with unfavorable outcomes, and compare the outcomes of death and the need for Acute Kidney Support Therapy (AKST) between patients with DI-AKI vs. AKI due to other etiologies.

**Methods:**

A retrospective cohort study conducted at the Hospital das Clínicas of the Faculty of Medicine of Botucatu – UNESP (HC-FMB), using data from patients hospitalized between January 2016 and April 2022 and followed, via consultation, by the AKI-Nephrology team. Inclusion criteria: diagnosis of AKI and Chronic Kidney Disease (CKD) with superimposed AKI. Exclusion criteria: patients under 18 years old or on chronic Renal Replacement Therapy. AKI was diagnosed based on creatinine increase as established by KDIGO 2012. Data were presented as mean and standard deviation or median with interquartile range and frequency. Statistical significance was set at 5% (*p* < 0.05). Comparative analyses were performed using the Chi-Square test for categorical variables and the T-test for continuous variables. Subsequently, logistic regression was performed to identify factors associated with the need for AKST and death.

**Results:**

A total of 1,398 patients were analyzed, most of them males (61.4%), with a mean age of 64 years ±14.4 years. The most prevalent etiology of AKI was Mixed Ischemic + Septic AKI (28%). DI-AKI was a significant cause of AKI (19.3%). Of these, 25.2% were isolated DI-AKI and 74.8% were Mixed DI-AKI + Ischemia and/or Sepsis. Among patients with DI-AKI, the mean age was 61.15 ± 15.26, males were the most frequent, the majority were not subjected to AKST and survived. Most of these patients were hospitalized in the ward, did not need vasoactive drugs, nor did they use mechanical ventilation. DI-AKI showed lower severity and mortality compared to other AKI etiologies but had a similar need for AKST (26.3% vs. 35.4%, *p* < 0.05 and 31.8% vs. 36.8%, *p* > 0.05). Most nephrotoxic drugs caused type A reactions, with Vancomycin being the primary nephrotoxin. Among drugs associated with DI-AKI, Vancomycin was associated with a higher need for AKST and death, while Amphotericin B was associated with a lower risk of AKST and death.

**Conclusion:**

Although the mortality rate is lower among DI-AKIs compared to other AKI etiologies, the need for AKST was similar. Therefore, it is recommended that DI-AKI be recognized early to enable dose reduction or even drug suspension, depending on the type of reaction, to reduce healthcare costs and improve clinical outcomes for patients.

## Introduction

Acute Kidney Injury (AKI) is a common clinical syndrome characterized by a sudden reduction in the glomerular filtration rate (GFR), causing severe alterations in blood volume and acid-base balance (1). Prompt identification of the cause of AKI and early measures to halt the progression of renal injury are known to drastically reduce its associated morbidity and mortality (1).

Drug-Induced Acute Kidney Injury (DI-AKI), a clinical entity associated with exposure to potentially nephrotoxic medications is particularly common among hospitalized patients (2). While exposure to nephrotoxic drugs is a requirement for the occurrence of DI-AKI, drug characteristics are not the only factors to contribute to nephrotoxicity (2). Underlying patient characteristics and kidney factors are also significant determinants (2).

Prolonged use of nephrotoxic medications or the combination of multiple nephrotoxic drugs are factors that predispose to a higher risk of renal injury. Insoluble drugs and metabolites with crystal precipitation also pose a higher risk of nephrotoxicity (2). Patients most predisposed to nephrotoxicity are elderly women (over 65 years), especially those with Chronic Kidney Disease (CKD) or cirrhosis (2). Additional risk factors include hypervolemic status, obstructive jaundice, and nephrotic syndrome (2). Kidney perfusion accounts for 25% of cardiac output, which leads to high drug concentrations in the kidney medulla and interstitium, favoring the accumulation of metabolites in these areas (2).

Although AKI in hospitalized patients is often multifactorial, studies indicate that DI-AKI accounts for 14 to 37% of AKI cases (3–8). No specific diagnostic markers for DI-AKI have been identified; thus, diagnosis requires considering other risk factors to establish an appropriate causal relationship (4).

Pathophysiologically, DI-AKI can occur through Acute Tubular Necrosis (ATN), Acute Interstitial Nephritis (AIN), and Crystal-Induced Nephropathy. ATN is characterized by drug-induced damage to the renal tubules, causing cellular apoptosis and subsequent AKI episodes (9). AIN is an immune-mediated reaction characterized by immune infiltration into tubulointerstitial cells. Protective factors against renal injury include avoiding supra-therapeutic doses of medications, concurrent use of nephrotoxic drugs, and preventing hypotensive episodes. In some cases of AIN, glucocorticoids are indicated due to the immune-mediated nature of the condition (9).

The rarest pathophysiological mechanism of DI-AKI is Crystal-Induced Nephropathy, characterized by intratubular crystal deposition seen in renal biopsy (9). Preventive measures are similar to those for other mechanisms, with additional steps to achieve the ideal urinary pH during the use of certain drugs to avoid crystal precipitation (9). Therapeutically, discontinuing the offending drug, avoiding new nephrotoxic drugs, and preventing hypovolemia are recommended (9).

A broader classification, including adverse drug reactions comprises type A and type B reactions. Type A reactions are predictable based on the pharmacology of the substance, dose-dependent, and manifest as ATN. Type B reactions are unpredictable, idiosyncratic, not dose-dependent, and manifest as AIN, and Crystal-Induced Nephropathy, among others (4).

Although DI-AKI is a significant cause of AKI among hospitalized patients, affecting morbidity and mortality, the medical literature remains insufficient regarding its epidemiology, pathophysiological mechanisms, and related prognostic factors. Therefore, the main objectives of this study was to evaluate the incidence of DI-AKI and identify the causative drugs and the pathophysiological mechanisms of the observed injury. Furthermore, secondary objectives were to establish the main prognostic factors associated with unfavorable outcomes and compare the outcomes of death and the need for Acute Kidney Support Therapy (AKST) between patients with DI-AKI and those with AKI of other etiologies.

## Patients and methods

A retrospective cohort study was conducted on patients hospitalized at Botucatu Medical School Hospital – UNESP (HC-FMB) between January 2016 and April 2022, who were followed by the AKI-Nephrology team. Clinical, laboratory, and epidemiological data were extracted from the DataIRA system. The STROBE method for cohort studies was followed as described Supplementary material (10).

Patients diagnosed with AKI and CKD with superimposed AKI were included. Exclusion criteria were age under 18 years and Chronic Kidney Replacement Therapy. According to the KDIGO 2012 criteria, AKI was diagnosed based on creatinine levels (≥0.3 g/dL within 48 h or 1.5x baseline within 7 days), and stratified into KDIGO AKI stages 1, 2, or 3, or CKD with superimposed AKI (11). The baseline creatinine levels for the diagnosis of AKI was considered based on the lowest creatinine levels in the last 6 months. According to the KDIGO 2013 criteria, CDK was diagnosed based on glomerular filtration rate < 60 mL/min/1.73m^2^ present for >3 months (12).

The criteria used to classify the etiology of AKI as drug-induced AKI were: (1) The duration of drug exposure must be at least 24 h and must precede the event; (2) There should be biological plausibility for the suspected drug to cause de kidney injury; (3) An assessment of the relative contribution of the suspected drug vs. concomitant risks and exposures to other nephrotoxic agents should be employed; (4) The strength of the relationship between the suspected drug and injury should be based on drug exposure, duration of therapy, and the temporal relationship (3).

Drug reactions were classified on the basis of their underlying pathophysiological mechanisms into type A and type B reactions. Type A drugs included those that can lead to ATN, such as Vancomycin, contrast agents, Aminoglycosides, and Amphotericin B. Type B drugs included those associated with AIN or crystal-induced nephropathy, such as Quinolones, Cephalosporins, Immunosuppressants, Acyclovir, Polymyxin B, and Non-steroidal anti-inflammatory drugs (NSAIDs) (13–15).

Non-drug-induced AKI etiologies were considered nephrotoxic due to endogenous toxins, ischemic, septic, mixed ischemic and septic, obstructive, and COVID-19 related. The diagnosis and etiology of AKI were defined by the clinical team based on clinical criteria and laboratory tests.

AKI diagnosis and etiology were defined by the clinical team based on clinical (insult identification) and laboratory (plasma and urine) criteria. The classification of type A and B reactions was performed by researchers based on pathophysiological mechanisms (13), the classification was revised by at least two researchers, to avoid bias.

Data collected from DataIRA included: full name, gender, age, date of hospitalization, date of nephrology evaluation, clinical summary, primary diagnosis, AKI etiology, AKI classification, involved drug (if drug-induced), serial serum creatinine levels, need for mechanical ventilation (MV), vasopressor, Acute Kidney Support Therapy (AKST), as well as outcome (discharge or death). Additionally, for each patient, the ATN-ISS (16) score was calculated to predict the probability of death in a patient with AKI.

The study was approved by the institutional Ethics Committee of Botucatu Medical School (CAAE 59643522.8.0000.5411).

### Data analysis

Data analysis was performed using STATA 8.0 (17). Data were presented as mean and standard deviation or median with interquartile range. Descriptive analysis was performed for all DI-AKI patients, calculating measures of central tendency and dispersion for continuous variables and frequency for categorical variables. Comparative analyses used AKST need, patient outcome (death), and AKI etiology as outcome variables. The Chi-Square test was used for comparing categorical variables. The t-test was used for continuous variables when the outcome variable was “need for AKST” or “death.” A multivariate analysis was conducted using logistic regression models, calculating Odds Ratios (OR). All independent variables associated with the outcomes of death and AKST need, with *p* ≤ 0.20, were included in the model. Statistical significance was set at *p* < 0.05. Data analysis was performed using SAS for Windows, version 9.2 (North Carolina, USA, 2009).

## Results

**Figure 1:**
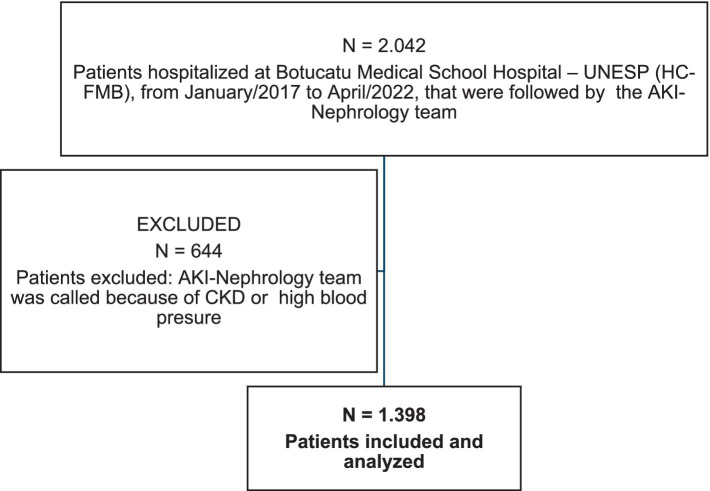
Flowchart of the selection and elimination of patients to complete the cohort.

There were 1,398 patients included in the study. The majority were male [*N* = 859 (61.4%)], with a mean age of 64 years ±14.4 years. The most prevalent etiologies of AKI were Mixed Ischemic AKI + Septic AKI (28%), Ischemic AKI (19.4%), Mixed DI-AKI + Ischemia and/or Sepsis (14.4%), and Septic AKI (10%), followed by DI-AKI (4.8%). Of the 1,398 patients studied, 600 (42.9%) were in the Intensive Care Unit (ICU), 580 (41.4%) required mechanical ventilation (MV), and 694 (49.6%) were on vasopressor. The mean ATN-ISS score was 0.5 ± 0.28. Acute Kidney Support Therapy (AKST) was needed by 35.8% of the patients. The most frequent AKI stage was KDIGO 3 (44.4%), followed by CKD with superimposed AKI (37.9%), KDIGO 2 (12.4%), and KDIGO 1 (5.1%). There were 471 (33.6%) deaths (Figure 1).

Among the 1,398 patients analyzed, 270 (19.3%) had DI-AKI. Of these, 25.2% had isolated DI-AKI (*N* = 68), and 74.8% had Mixed DI-AKI + Ischemia and/or Sepsis (*N* = 202). The mean age of patients with DI-AKI was 61.15 ± 15.26 years, with males being more frequent (62.9%). Most of them did not require AKST (68.2%) and survived (73.7%). The majority were hospitalized in wards (63.7%), did not need vasopressor (56%), and did not use MV (63.8%). The most frequent AKI stage was KDIGO 3 (47.4%), followed by CKD with superimposed AKI (28.8%), KDIGO 2 (18.5%), and KDIGO 1 (5.1%). Type A reactions predominated (77%) over type B reactions (23%). The most frequently identified mechanism was ATN (77%), followed by AIN (15.2%) and Crystal-Induced Nephropathy (2.6%), as shown in Table 1.

**Table 1:** Clinical and demographic characteristics of patients with DI-AKI included in the study, comparison of clinical characteristics and outcomes between patients with type A and B drug reactions and classification of DI-AKI according to the type of reaction and pathophysiological mechanism.

	DI-AKI (*N* = 270) (%)	Type A reactions (*N* = 208) (77%)	Type B reactions (*N* = 62) (23%)	*p*
Male	170 (62.9)	134 (64.4)	36 (58)	0.18
Age (in years)	61.15 ± 15.26	61.2 ± 15.26	65.1 ± 16.8	0.05
DI-AKI	68 (25.2)	56 (26.9)	12 (19.3)	0.12
KDIGO 3	128 (47.4)	101 (48.5)	28 (45.1)	0.44
AKST	86 (31.8)	67 (32.2)	19 (30.6)	0.66
ICU	98 (36.2)	83 (39.9)	15 (24.1)	<0.001
Vasopressor	119 (44)	98 (47.1)	20 (32.2)	0.01
MV	98 (36.2)	86 (41.3)	12 (19.3)	0.001
ATN-ISS	0.39 ± 0.29	0.41 ± 0.29	0.36 ± 0.27	0.08
Deaths	71 (26.2)	58 (27.8)	13 (20.9)	0.22
Pathophysiological mechanism				
ATN	208 (77)			
AIN	41 (15.2)			
Crystal-induced nephropathy	7 (2.6)			
Others	14 (5.2)			

DI-AKI, Drug-Induced Acute Kidney Injury; KDIGO, Kidney Disease: Improving Global Outcomes (11); AKST, Acute Kidney Support Therapy; ICU, Intensive Care Unit; MV, Mechanical Ventilation; ATN-ISS, ATN-ISS (16) score; ATN, Acute Tubular Necrosis; AIN, Acute Interstitial Nephritis.

Table 1 also compares the clinical characteristics and outcomes between patients with type A and B drug reactions. Both types were similarly associated with AKST need, mortality, and ATN-ISS score. They commonly led to KDIGO 3 AKI, had a similar prevalence of isolated DI-AKI, and were more common in males. However, they differed in age and the need for vasopressor, MV, and ICU stay. Type A reactions affected younger patients more frequently and were more common among ICU patients requiring vasopressor and MV.

**Figure 2:**
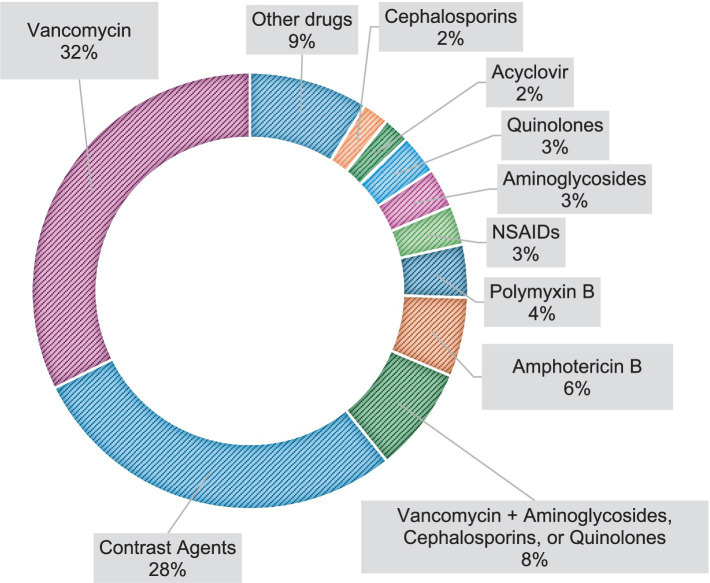
Most prevalent drugs involved in DI-AKI in the studied population.

The most prevalent drugs involved in DI-AKI, in order of incidence, were Vancomycin, contrast agents, Vancomycin + Aminoglycosides, Cephalosporins, or Quinolones, Amphotericin B, Polymyxin B, NSAIDs, Aminoglycosides, Quinolones, Acyclovir, and Cephalosporins, as described in Figure 2.

Patients with DI-AKI were compared for AKST need and death outcomes, as described in Tables 2, 3. Factors associated with death and AKST need included MV use, higher ATN-ISS, and Vancomycin use. Mixed etiology (DI-AKI + Ischemia and/or Sepsis) was associated with higher AKST need, while Amphotericin B-related AKI was associated with lower AKST need.

**Table 2:** Factors associated with death among patients with DI-AKI in univariate analysis and logistic regression.

	Death (*N* = 69) (%)	Alive (*N* = 201) (%)	Univarite analysis *p* value	Multivariate analysis OR (95% IC); *p* value
Age (in years)	62.4 ± 12.4	60.69 ± 16.14	0.41	
MV	53 (76.8)	46 (22.8)	< 0.001	1.34 (1.12–4.99); 0.049
ATN-ISS	0.61 ± 0.27	0.31 ± 0.26	< 0.001	5.78 (0.98–34); 0.052
Vancomycin	30 (43.4)	58 (28.8)	<0.001	6.69 (1.21–13.52); 0.021
Vasopressor	59 (85.5)	62 (30.8)	< 0.001	
ICU	46 (66.6)	51 (25.3)	< 0.001	
Mixed DI-AKI + Ischemia and/or Sepsis	57 (82.6)	141 (70.1)	0.06	
Contrast agents	19 (27.5)	57 (28.3)	0.85	
Amphotericin B	0 (0)	15 (7.4)	0.04	
Acyclovir	1 (1.4)	5 (2.4)	0.71	
Quinolones	1 (1.4)	6 (2.9)	0.65	
Aminoglycosides	5 (7.2)	6 (2.9)	0.23	
NSAIDs	0 (0)	9 (4.4)	0.45	
Polymyxin B	2 (2.8)	10 (4.9)	0.49	

MV, Mechanical Ventilation; ICU, Intensive Care Unit; ATN-ISS, ATN-ISS (16) score; DI-AKI, Drug-Induced Acute Kidney Injury; NSAIDs, Non-steroidal anti-inflammatory drugs.

**Table 3:** Factors associated with AKST among patients with DI-AKI in univariate analysis and logistic regression.

	AKST (*N* = 81) (%)	Non AKST (*N* = 189) (%)	Univarite analysis *p* value	Multivariate analysis OR (95% IC); *p* value
Age (in years)	61.52 ± 13.76	62.56 ± 15.64	1.0	
MV	61 (75.3)	39 (20.6)	< 0.001	1.33 (1.12–2.92); 0.034
ATN-ISS	0.59 ± 0.27	0.31 ± 0.26	< 0.001	5.51 (1.03–29.52); 0.046
Vancomycin	36 (44.4)	52 (27.5)	0.03	1.67 (1.07–5.9); 0.025
Amphotericin B	1 (1.2)	14 (7.4)	0.08	0.086 (0.81–0.99); 0.031
Mixed DI-AKI + Ischemia and/or Sepsis	70 (86.4)	126 (66.6)	0.01	1.09 (1.04–1.87); 0.041
Vasopressor	66 (81.4)	53 (28)	< 0.001	
ICU	52 (64.1)	42 (22.2)	< 0.001	
Contrast agents	20 (24.6)	57 (27.5)	0.34	
Acyclovir	0 (0)	6 (3.1)	0.24	
Quinolones	3 (3.7)	4 (2.1)	0.77	
Aminoglycosides	7 (8.6)	4 (2.1)	0.03	
NSAIDs	1 (1.2)	8 (4.2)	0.45	
Polymyxin B	1 (1.2)	11 (5.8)	0.13	

MV, Mechanical Ventilation; ICU, Intensive Care Unit; ATN-ISS, ATN-ISS (16) score; DI-AKI, Drug-Induced Acute Kidney Injury; NSAIDs, Non-steroidal anti-inflammatory drugs.

Finally, the study population was compared for death and AKST need according to AKI etiology (DI-AKI vs. non-drug-induced AKI). Non-drug-induced AKI showed higher severity and mortality compared to DI-AKI; however, no difference was found in AKST need between the two groups (Table 4).

**Table 4:** Comparison of DI-AKI vs. Non-drug-induced AKI groups regarding clinical characteristics and outcomes.

	Non-drug-induced AKI (*N* = 1,128) (%)	DI-AKI (*N* = 270) (%)	*p*
Male	672 (59.7)	163 (60.3)	0.98
Age (in years)	64.1 ± 15.8	61.15 ± 15.8	0.01
KDIGO 3	501 (44.4)	122 (45.1)	0.78
AKST	415 (36.8)	86 (31.8)	0.14
ICU	502 (44.5)	98 (36.2)	0.015
Vasopressor	595 (52.7)	99 (36.6)	0.001
MV	468 (41.4)	102 (37.7)	0.22
ATN-ISS	0.56 ± 0.29	0.36 ± 0.27	< 0.001
Deaths	400 (35.4)	71 (26.3)	0.005

KDIGO, Kidney Disease: Improving Global Outcomes (9); AKST, Acute Kidney Support Therapy; ICU, Intensive Care Unit; MV, Mechanical Ventilation; ATN-ISS: ATN-ISS (13) score.

## Discussion

This study evaluated over 1,390 patients presenting in-hospital AKI. When comparing DI-AKI with AKI due to other etiologies, DI-AKI showed lower mortality but the same rate of AKST need (26.3% vs. 35.4%, *p* < 0.05, and 31.8% vs. 36.8%, *p* > 0.05), probably because it affects patients in less severe conditions, i.e., fewer ICU admissions, and less frequent need for vasopressor and MV. DI-AKI is considered reversible and may be avoided by monitoring kidney function, measuring drug serum levels, and keeping a high level of suspicion when potentially nephrotoxic drugs are used. DI-AKI is associated with significant morbidity but lower mortality than AKI due to other etiologies. However, as it is associated with a similar AKST rate, it also imposes high healthcare costs.

In the last decades, there has been an increase in the incidence of AKI which has now become a common complication among ICU patients. It is associated with worse outcomes such as longer hospital stays, development of CKD, and increased short- and long-term mortality risk (8). Assessing DI-AKI causality is often complicated by the presence of competing risk factors that bring uncertainty about the nephrotoxic risk of commonly used medications (3).

The prevalence of DI-AKI in our study was 19.3% and type A reactions were the most observed. The drugs most associated with DI-AKI were Vancomycin, contrast agents, Amphotericin B, Polymyxin B, NSAIDs, aminoglycosides, quinolones, acyclovir, and cephalosporins.

Vancomycin was the drug most associated with DIAKI in our study. In the last few years, the association between vancomycin and anti-pseudomonas antibiotics has drawn attention, especially when combined with piperacillin-tazobactam. One cohort study result showed that vancomycin + piperacillin-tazobactam increases the risk of AKI and AKST when compared with both vancomycin + meropenem and vancomycin + cefepime (18). Another cohort showed the opposite: vancomycin + piperacillin-tazobactam was not associated with a greater risk of AKI and AKST than other broad-spectrum combinations (19). In most series it did not increase the risk of AKI stage 2 or 3, AKST or mortality, which led to speculations if vancomycin + piperacillin-tazobactam is DIAKI ou pseudoAKI – whereby AKI stage 1 could be attributable to an impairment in creatine secretion (20, 21).

A multicenter observational study in South Africa identified a 37% prevalence of DI-AKI among hospitalized patients, with the main causative drugs being contrast agents, tenofovir, and aminoglycosides (7). Similarly, a study in China reported a comparable prevalence of DI-AKI, with diuretics, proton pump inhibitors, and antibiotics being the most associated drugs (6). The prevalence found in our study is lower compared to African and Chinese data, but contrast agents and antibiotics were also causative drugs among our patients.

Contrast agents were the second drug most associated with DIAKI in our study, in literature, it is recognized as the third leading cause of in-hospital AKI (22). The two main risk factors are CKD and diabetes mellitus (22). One strategy known to reduce the risk of contrast-induced nephropathy in patients with CKD is sodium bicarbonate, although it does not reduce mortality or the risk of AKST (22, 23).

In a 2018 retrospective analysis by Welch et al., based on the United States Food and Drug Administration Adverse Event Reporting System (FAERS), the main drugs associated with DI-AKI were aprotinin, sodium phosphate, furosemide, vancomycin, and metformin (24). In our study, although diuretics were not associated with DI-AKI, vancomycin was one of most frequently associated drugs.

Another multicenter study, including data mainly from hospitals in Europe and North America, identified a prevalence of around 14% for DI-AKI, with diuretics and NSAIDs as the main associated drugs (8). These data align with our prevalence findings but differ in the most frequently associated drugs.

Among patients with DI-AKI, 31.8% required AKST, and 26.3% died. Similar data were reported in a prospective cohort study in Shanghai, which identified a 28.9% prevalence of DI-AKI, a 34.3% rate of AKST requirement, and a 28.8% mortality rate (5). In our study, although prevalence was lower (19.3% vs. 28.9%), the mortality and AKST need rates were similar.

In our patients with DI-AKI, logistic regression revealed that AKST requirement was associated with the need for MV, higher ATN-ISS, and mixed etiology (DI-AKI + Ischemia and/or Sepsis), with MV use and higher ATN-ISS also associated with death. Additionally, vancomycin was associated with AKST need and death, while amphotericin B was associated with a lower rate of AKST requirement.

Our results show that, consistently with other studies on AKI due to other etiologies, MV use was associated with unfavorable outcomes, especially because such patients are often in ICU and often on vasopressors (25). Vancomycin was associated with poorest clinical outcomes, leading to type A reactions, which are dose and time-dependent and can be avoided by reducing the dose or exposure or limiting the exposure time rather than completely discontinuing the medication (13).

In 2017, Awdishu et al. (4) proposed a new tool to address drug-induced nephrotoxicity focusing on risk assessment, early recognition, targeted response, timely renal support, rehabilitation, and research: the 6R approach. Risk assessment should consider previous kidney function, the patient’s current clinical condition, and the known nephrotoxicity of the drug. DI-AKI recognition should be as early as possible, and the appropriate response will vary depending on the drug used. Although the rate of AKST need may be low, hemodialysis can reduce drug toxicity when certain drugs such as vancomycin and aminoglycosides are used (4).

Typically, DI-AKI is non-oliguric and resolves with drug discontinuation. However, monitoring these patients in AKI outpatient clinics is recommended to identify cases where kidney function recovery is delayed. Documenting previous nephrotoxicity in patient charts can reduce re-exposure to the same drug (4). Monitoring serum drug concentration can reduce DI-AKI prevalence and healthcare costs as well (26, 27).

Standardized definitions of DI-AKI are scarce, leading to challenges in recognizing, assessing the prevalence and the outcomes of DI-AKI. More research is needed on predictive analyses capable of identifying individuals at higher risk of nephrotoxicity (4).

Study limitations included its retrospective, single-center nature. The retrospective analysis and data collection from medical records depend on the accurate recording by healthcare professionals. Besides retrospective, it is a single-center study, and including other centers could contribute to a broader patient profile and better result reproducibility. The duration of drug use, vancomycin serum concentration monitoring, and the administered contrast route were not evaluated. Despite these limitations, our results suggest that DI-AKI is a frequent AKI etiology, mainly caused by vancomycin (a type A reaction and potentially preventable), with high mortality rates, although lower than other AKI etiologies but with the same rate of AKST need.

## Conclusion

DI-AKI was a significant cause of AKI (19.3%), showing lower severity and mortality but similar rate of AKST need compared to AKI due to other etiologies. Most nephrotoxic drugs caused type A reactions, with vancomycin being the primary nephrotoxin, suggesting that drug-induced nephrotoxicity can be avoided as a cause of AKI, reducing healthcare costs and improving patient outcomes. Before using drugs with high nephrotoxic potential, it is recommended to assess the patient’s risk factors and, should DI-AKI occur, ensure early recognition and appropriate rehabilitation. Further research is required for the assessment of causality and development of predictive analyses on nephrotoxicity risk, and consequently reduce the healthcare costs associated with DI-AKI.

## Data Availability

The raw data supporting the conclusions of this article will be made available by the authors, without undue reservation.
