# Supplementary material for: Drug-induced acute kidney injury: a cohort study on incidence, identification of pathophysiological mechanisms, and prognostic factors
Source: Front Med (Lausanne). 2024 Oct 29;11:1459170. doi: 10.3389/fmed.2024.1459170 (PMC11554514; doi:10.3389/fmed.2024.1459170)
Supplement: Supplementary file 1 [file Data_Sheet_1.PDF]

STROBE Statement—checklist of items that should be included in reports of observational studies

	Item No.	Recommendation	Page No.	Relevant text from manuscript
**Title and abstract**	1	(*a*) Indicate the study's design with a commonly used term in the title or the abstract	1	"A Cohort Study…"
		(*b*) Provide in the abstract an informative and balanced summary of what was done and what was found	1	"Methods" section of the abstract
**Introduction**				
Background/rationale	2	Explain the scientific background and rationale for the investigation being reported	2-3	3rd to 7th paragraphs of "Introduction" section
Objectives	3	State specific objectives, including any prespecified hypotheses	3	8th paragraph of "Introduction" section
**Methods**				
Study design	4	Present key elements of study design early in the paper	4	1st paragraph of "patients and methods" section
Setting	5	Describe the setting, locations, and relevant dates, including periods of recruitment, exposure, follow-up, and data collection	4 - 5	1st to 6th paragraph of "patients and methods" section
Participants	6	Give the eligibility criteria, and the sources and methods of selection of participants. Describe methods of follow-up	4 - 5	2nd to 6th paragraph of "patients and methods" section
Variables	7	Clearly define all outcomes, exposures, predictors, potential confounders, and effect modifiers. Give diagnostic criteria, if applicable	4	2nd to 4th paragraph of "patients and methods" section
Data sources/ measurement	8*	For each variable of interest, give sources of data and details of methods of assessment (measurement). Describe comparability of assessment methods if there is more than one group	4	5th paragraph of "patients and methods" section
Bias	9	Describe any efforts to address potential sources of bias	4	5th paragraph of "patients and methods" section
Study size	10	Explain how the study size was arrived at	6	Figure 1

Continued on next page

Quantitative variables	11	Explain how quantitative variables were handled in the analyses. If applicable, describe which groupings were chosen and why	5	"Data analysis" section
Statistical methods	12	(*a*) Describe all statistical methods, including those used to control for confounding	5	"Data analysis" section
		(*b*) Describe any methods used to examine subgroups and interactions	5	"Data analysis" section
		(*c*) Explain how missing data were addressed	-	
		(*d*) *Cohort study*—If applicable, explain how loss to follow-up was addressed	-	
		(*e*) Describe any sensitivity analyses	5	"Data analysis" section
**Results**				
Participants	13*	(a) Report numbers of individuals at each stage of study—eg numbers potentially eligible, examined for eligibility, confirmed eligible, included in the study, completing follow-up, and analysed	6	Figure 1
		(b) Give reasons for non-participation at each stage	6	Figure 1
		(c) Consider use of a flow diagram	6	Figure 1
Descriptive data	14*	(a) Give characteristics of study participants (eg demographic, clinical, social) and information on exposures and potential confounders	6 - 7	1st and 2nd paragraph of "Results" section
		(b) Indicate number of participants with missing data for each variable of interest	-	
		(c) *Cohort study*—Summarise follow-up time (eg, average and total amount)	7 - 8	Table 1
Outcome data	15*	*Cohort study*—Report numbers of outcome events or summary measures over time	8 - 11	Table 1
Main results	16	(*a*) Give unadjusted estimates and, if applicable, confounder-adjusted estimates and their precision (eg, 95% confidence interval). Make clear which confounders were adjusted for and why they were included	9 - 10	Tables 2 and 3
		(*b*) Report category boundaries when continuous variables were categorized	-	
		(*c*) If relevant, consider translating estimates of relative risk into absolute risk for a meaningful time period	-	

Continued on next page

Other analyses	17	Report other analyses done—eg analyses of subgroups and interactions, and sensitivity analyses	9 – 10	Tables 2 and 3
**Discussion**				
Key results	18	Summarise key results with reference to study objectives	12 - 14	1st to 10th paragraph of "Discussion" section
Limitations	19	Discuss limitations of the study, taking into account sources of potential bias or imprecision. Discuss both direction and magnitude of any potential bias	14	Last paragraph of "Discussion" section
Interpretation	20	Give a cautious overall interpretation of results considering objectives, limitations, multiplicity of analyses, results from similar studies, and other relevant evidence	12 - 14	1st to 10th paragraph of "Discussion" section
Generalisability	21	Discuss the generalisability (external validity) of the study results	15	"Conclusion"
**Other information**				
Funding	22	Give the source of funding and the role of the funders for the present study and, if applicable, for the original study on which the present article is based	-	

Note: An Explanation and Elaboration article discusses each checklist item and gives methodological background and published examples of transparent reporting. The STROBE checklist is best used in conjunction with this article (freely available on the Web sites of PLoS Medicine at http://www.plosmedicine.org/, Annals of Internal Medicine at http://www.annals.org/, and Epidemiology at http://www.epidem.com/). Information on the STROBE Initiative is available at www.strobe-statement.org.
